# Visible feature engineering to detect fraud in black and red peppers

**DOI:** 10.1038/s41598-024-76617-1

**Published:** 2024-10-25

**Authors:** Mohammad Hossein Nargesi, Kamran Kheiralipour

**Affiliations:** Mechanical Engineering of Biosystems Department, Ilam University, Ilam, Iran

**Keywords:** Fraud detection, Image processing, Feature engineering, Artificial intelligence., Engineering, Materials science

## Abstract

Visible imaging is a fast, cheap, and accurate technique in the assessment of food quality and safety. The technique was used in the present research to detect sea foam adulterant levels in black and red peppers. The fraud levels included 0, 5, 15, 30, and 50%. Sample preparation, image acquisition and preprocessing, and feature engineering (feature extraction, selection, and classification) were the conducted steps in the present research. The efficient features were classified using artificial neural networks and support vector machine methods. The classifiers were evaluated using the specificity, sensitivity, precision, and accuracy metrics. The artificial neural networks had better results than the support vector machine method for the classification of different adulterant levels in black pepper with the metrics’ values of 98.89, 95.67, 95.56, and 98.22%, respectively. Reversely, the support vector machine method had higher metrics’ values (99.46, 98.00, 97.78, and 99.11%, respectively) for red pepper. The results showed the ability of visible imaging and machine learning methods to detect fraud levels in black and red pepper.

## Introduction

Food safety is an important issue in line with the continuous improvement of living standards^1,2^. Quality inspection is an important element in the technical aspect of sustainable production^3^. One of the problems affecting food quality is adulteration. Adulteration is a serious problem in food safety which reduces the quality of products^4–6^. It includes mislabeling, dilution, unauthorized improvements, substitution, concealment, and counterfeiting^7,8^. Adulterants are low-quality substances added to different products to increase incomes^9,10^.

Different adulterants have been detected in milk^11,12^, saffron^13^, olive oil^14^, and honey^15^. Accurate laboratory methods such as HPLC-based chemometric analysis^16^, NIR and FT-IR^17^, GC×GC-MS^18^, and SPME-GC-HRMS^19^ have been applied to detect adulterations in food. These methods are time-consuming, expensive, and require a precise laboratory and a skilled operator. Therefore, it is necessary to use cheap and fast methods that do not require a skilled operator. So, the novel goal of the present research is the detection of fraud using an automatic, simple, and low-cost method.

Imaging is a fast, accurate, and cheap technology that includes different visible and invisible techniques^20–22^. Due to high abilities and valuable advantages, the technology has widely been used to detect different goals^23–27^. It has been used for assessing morphology, color, and textural features of different materials to detect external and internal goals^28–33^. In detecting adulteration in food rapeseed and sunflower oils in olive oil have been detected using image processing^34^. However, the method has been used to detect other frauds such as vegetable oil adulterants in olive oil^35–37^, different kinds of adulterants in rice^38,39^, melamine and diluting in milk^40,41^, different adulterants in pistachios and mung beans^40,42^ and fraud in turmeric powder^43^. So, the novel goal of the present research is to develop a visible image processing algorithm to detect sea foam adulteration in black and red pepper.

Pepper is one of the main food additives in the world^44^. All over the world, pepper is cultivated with different types and varieties that have different yields and levels of spiciness^45^. Red (Capsicum annum) and black (Piper nigrum) pepper are different pepper types that have been studied in the present research. Different adulterants such as salt, wheat flour, wheat bran, rice bran, and Sudan dye have been detected in red pepper because it is a cheap material and makes more profit^46,47^.

Machine learning includes different trained-based methods are applied to analyze images’ data^48–50^. The ability of online applications and high accuracy caused vast applications of the methods in different fields. These methods have been widely used to classify extracted features from the images of different objects^51–53^. So, artificial neural networks and support vector machines as the most famous learning-based classification methods have been used in the present research.

As fraud affects consumers and endangers their health, detection of food fraud is necessary to ensure the legitimate interests of consumers. On the other side, laboratory evaluation of food fraud is usually low precision, time-consuming, expensive, offline, and requires expert operators and in some cases needs referenced materials. So, the goal of the present research was to combine image processing and machine learning methods to detect sea foam adulterant levels in red and black pepper.

## Materials and methods

**Fig. 1:**
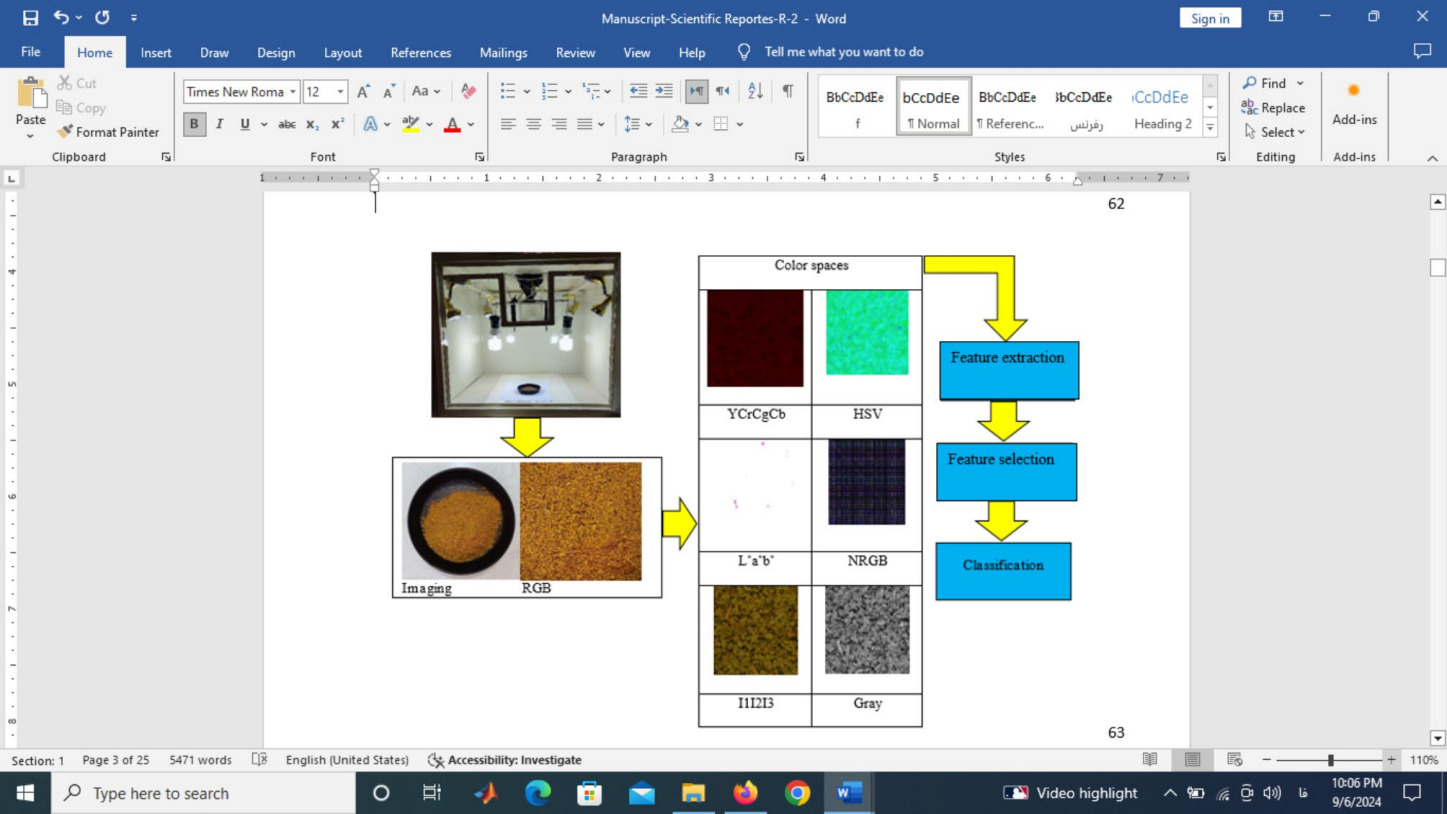
Imaging and image processing steps in the detection of sea foam levels in pepper.

Different steps in the present research were sample preparation, image acquisition and processing, and feature engineering (feature extraction, selection, and classification) (Fig. 1).

### Sample preparation

Original black and red peppers were purposed from the local markets in Ilam, Iran. The materials were ground to make powdered spices. Sea foam was used as adulterant material. It was powdered to be mixed with the based materials. The adulterant was added to the based materials to obtain 0, 5, 15, 30, and 50% fraud levels. All samples were carefully stored at 25 °C and away from light until imaging.

### Image acquisition

An imaging system was used to acquire the visible images of the prepared samples (Fig. 1). In the system, A Xiaomi Note 10 Pro Max camera was used to capture the images of the samples in a lighting box. The distance between the camera lens and the sample inside the the box was maintained 15 cm. Digital RGB images were captured and transported to a personal computer. In total, 180 images were obtained for the five fraud classes.

### Image processing

An algorithm was designed for processing the images of different samples. The algorithm was coded in MATLAB R2016b software.

#### Pre-processing

**Fig. 2:**
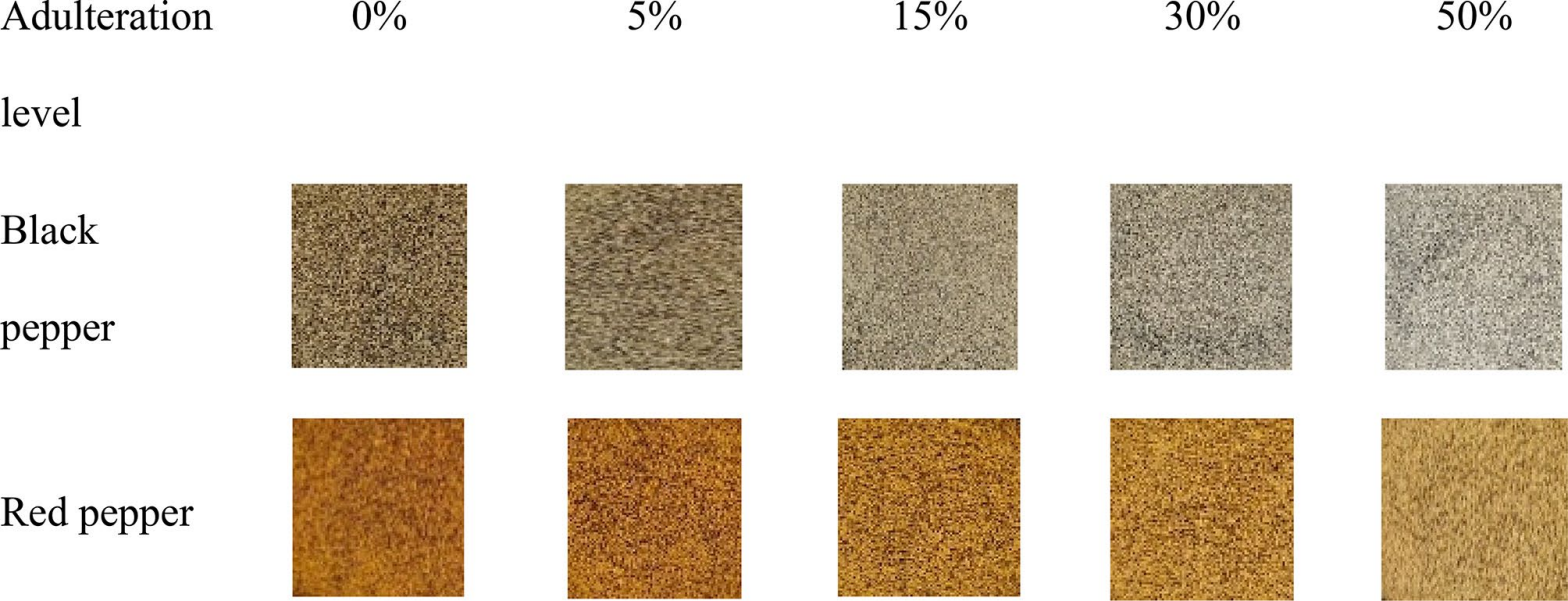
The cropped samples’ images.

Image preprocessing is the first stage of image processing that improves the images and or extracts useful areas from the images of interest^22,54^. In the present research, the obtained images were cropped to process the center area of the samples’ images (Fig. 2).

Digital RGB images were used to obtain L*a*b*, HSV, NRGB, CrCgCb, I1I2I3, and gray color spaces^22,53,55^. After obtaining the images in different color spaces, 19 different channels were extracted from the images including: R, G, B, L*, a*, b*, H, S, V, NR, NG, NB, Cr, Cg, Cb, I1, I2, I3 and gray^56–58^.

#### Feature extraction

In this step, the color and textural features from the obtained channels were extracted. Color features included average, minimum, maximum, median, mode, standard deviation, coefficient of variation, skewness, and kurtosis^3,59,60^. Energy, entropy, contrast, correlation, and homogeneity were extracted as textural features^58,59^. According to 19 image channels and 14 features for each channel, 266 features were extracted in total.

#### Feature selection

Since 266 features were extracted for each image, the classification of these features is a time-consuming task and may lead to a decrease in its accuracy. Among them, a few features were selected as efficient ones. To select the efficient features, the sequential feature selection method was used. The method finds efficient features based on the fit deviation criterion (a generalization of the residual sum of squares)^61^. To this end, an algorithm was designed and programmed in MATLAB software. In the developed algorithm, the input data was the extracted features and the target was the studied classes. The output of the algorithm after running, was a set of the efficient features.

### Classification

To categorize different fraud levels, the efficient features were classified. The efficient features were classified by artificial neural networks (ANN)^45,62,63^ and support vector machine (SVM)^64^.

In the classification using the ANN method, the Levenberg-Marquardt algorithm was applied for training the networks. The activation function of both the hidden and output layers was tansig^48,58^. To obtain the optimal network for distinguishing different classes of frauds in black pepper, different artificial neural network structures were evaluated. For this purpose, the number of neurons in the hidden layer was changed in the structures. In these structures, the number of neurons in the input layer was equal to the number of efficient features and the number of neurons in the output layer was equal to the number of fraud levels. The network was of the backpropagation type with feedforward, and the Levenberg-Marquardt training algorithm was used due to its high speed and also to prevent overtraining of the network. The data was divided to obtain different independent datasets. For the ANN method, three independent datasets were obtained as training, validation, and testing datasets that included 60, 20, and 20% of the whole data, respectively. The data was divided randomly to provide the three sets. As the three data sets were independent, the test data was used to validate the classifiers.

For the SVM method, 80 and 20% of the data were randomly selected to obtain independent training and testing datasets, respectively. As the two data sets were independent, the test data was used to validate the classifier. The SVM algorithm had a Gaussian kernel. Lambda, verbose, and c parameters of the models were equal to 1e-7, 1, and 1000, respectively. In this method, one-to-one strategy was used to classify the data.

In ANN, validation performance, overall correlation, and overall correct classification rate of different structures were considered to evaluate the models. For SVM, just overall correct classification rate of the method was calculated. Then different parameters for the models were calculated^11,30,57^:

1 $$\:\text{A}\text{c}\text{c}\text{u}\text{r}\text{a}\text{c}\text{y}=\frac{{\text{N}}_{\text{T}\text{P}}+{\text{N}}_{\text{T}\text{N}}}{{\text{N}}_{\text{T}\text{P}}+{\text{N}}_{\text{T}\text{N}}+{\text{N}}_{\text{F}\text{P}}+{\text{N}}_{\text{F}\text{N}}}\times\:100$$

2 $$\:\text{P}\text{r}\text{e}\text{c}\text{i}\text{s}\text{i}\text{o}\text{n}=\frac{{\text{N}}_{\text{T}\text{P}}}{{\text{N}}_{\text{T}\text{P}}+{\text{N}}_{\text{F}\text{P}}}\times\:100$$

3 $$\:\text{S}\text{e}\text{n}\text{s}\text{i}\text{t}\text{i}\text{v}\text{i}\text{t}\text{y}=\frac{{\text{N}}_{\text{T}\text{P}}}{{\text{N}}_{\text{T}\text{P}}+{\text{N}}_{\text{F}\text{N}}}\times\:100$$

4 $$\:\text{S}\text{p}\text{e}\text{c}\text{i}\text{f}\text{i}\text{c}\text{i}\text{t}\text{y}=\frac{{\text{N}}_{\text{T}\text{N}}}{{\text{N}}_{\text{T}\text{N}}+{\text{N}}_{\text{F}\text{P}}}\times\:100$$

where N_TP_ is the number of true classified positive samples, N_FP_ is the number of false classified positive samples, N_TN_ is the number of true classified negative samples, and N_FP_ is the number of false classified negative samples.

## Results and discussion

### Efficient features

Based on the feature selection task, 17 and 18 efficient features were obtained for black and red pepper, respectively (Tables 1 and 2). According to the results of Table 1, it can be seen that the values of the efficient features for black pepper were different for different fraud levels. As the percentage of fraud increased, the difference between the values of the effective features at different levels of fraud increased. The values of effective features for 50% fraud levels were higher for almost all features except for kurtosis of blue, median of b^*^, median of i2, mean of Cr, entropy f Cr, mean of saturation, standard deviation, median, and contrast of saturation channel.

**Table 1:** The mean values of the efficient features of different fraud levels of black pepper.

Feature	Channel	Adulteration level				
		0%	5%	15%	30%	50%
Mean	Gray	0.47	0.48	0.52	0.58	0.67
Mean	Red	0.55	0.55	0.58	0.62	0.69
Mean	Green	0.46	0.47	0.51	0.57	0.67
Mean	Blue	0.33	0.35	0.41	0.49	0.61
Kurtosis	Blue	4.88	3.95	3.97	4.35	4.21
Median	b*	16.62	15.31	11.84	7.89	5.18
Correlation	Nb	-0.11	-0.05	-0.08	-0.06	-0.03
Mean	i1	0.45	0.46	0.50	0.56	0.66
Median	i2	0.11	0.10	0.08	0.06	0.04
Mean	Cr	0.07	0.07	0.05	0.04	0.02
Entropy	Cr	4.09	4.17	3.89	3.70	3.03
Mean	Hue	0.10	0.10	0.10	0.11	0.12
Contrast	Hue	0.04	0.07	0.07	0.10	0.12
Mean	Saturation	0.48	0.45	0.35	0.23	0.14
Standard deviation	Saturation	0.23	0.24	0.18	0.13	0.06
Median	Saturation	0.38	0.36	0.28	0.19	0.12
Contrast	Saturation	2.10	2.47	1.58	0.76	0.26

According to the results of Table 2, it can be seen that the values of the efficient features of red pepper were different for the five fraud levels. The values of efficient features for 50% fraud levels were higher for almost all features except for mean of a*, median of a*, and median of b* that their values were highest for pure black pepper.

**Table 2:** The mean of the efficient features of different fraud levels of red pepper.

Feature	Channel	Adulteration level				
		0%	5%	15%	30%	50%
Mean	Gray	0.41	0.42	0.45	0.52	0.59
Entropy	Gray	7.48	7.51	7.57	7.56	7.49
Mode	Gray	0.06	0.05	0.05	0.05	0.07
Mean	Red	0.62	0.62	0.63	0.68	0.72
Mean	Green	0.37	0.38	0.42	0.49	0.57
Entropy	Green	7.31	7.39	7.51	7.57	7.53
Median	Green	0.40	0.42	0.46	0.55	0.63
Median	Blue	0.05	0.06	0.12	0.23	0.38
Max	L*	94.36	94.85	96.03	97.00	98.15
Mean	a*	20.58	18.78	15.87	11.55	6.94
Median	a*	14.71	13.22	10.70	8.01	5.03
Median	b*	70.01	66.23	54.70	40.39	26.92
Correlation	Nr	-0.15	-0.13	-0.11	-0.10	-0.06
Entropy	i1	7.32	7.36	7.45	7.47	7.45
Median	i1	0.37	0.39	0.42	0.51	0.59
Homogeneity	i2	0.90	0.91	0.92	0.93	0.99
Variation coefficient	Cr	0.14	0.14	0.13	0.11	0.13
Median	Cr	0.21	0.20	0.18	0.16	0.12

### Classification

#### ANN classifier

**Fig. 3:**
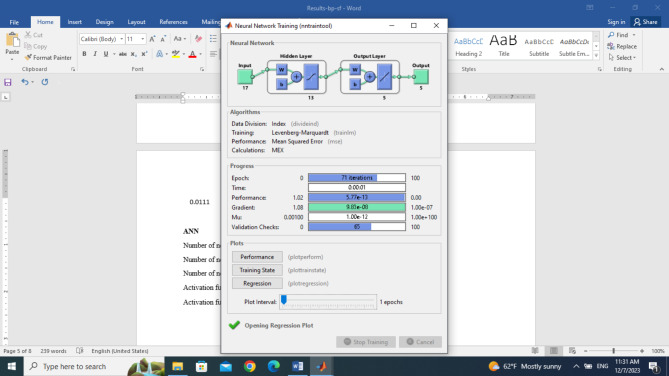
Optimal artificial neural network structure for classification of different levels of sea foam fraud in black pepper.

In the classification of different samples of black pepper adulterated with sea foam using the ANN method, different structures were evaluated from 17-1-5 to 17-20-5, and the best one was selected based on validation performance, overall correlation, and overall correct classification rate. The number of neurons in the input layer of the best model was equal to 17 (the number of efficient features) and the number of neurons in the output layer was 5 (different levels of fraud) Fig. 3. The highest percentage of the correct classification rate was obtained for a network with 17-13-5 structure. the number of neurons in the hidden layer was equal to 13. The images related to the optimal structure have been shown in Fig. 3.

**Fig. 4:**
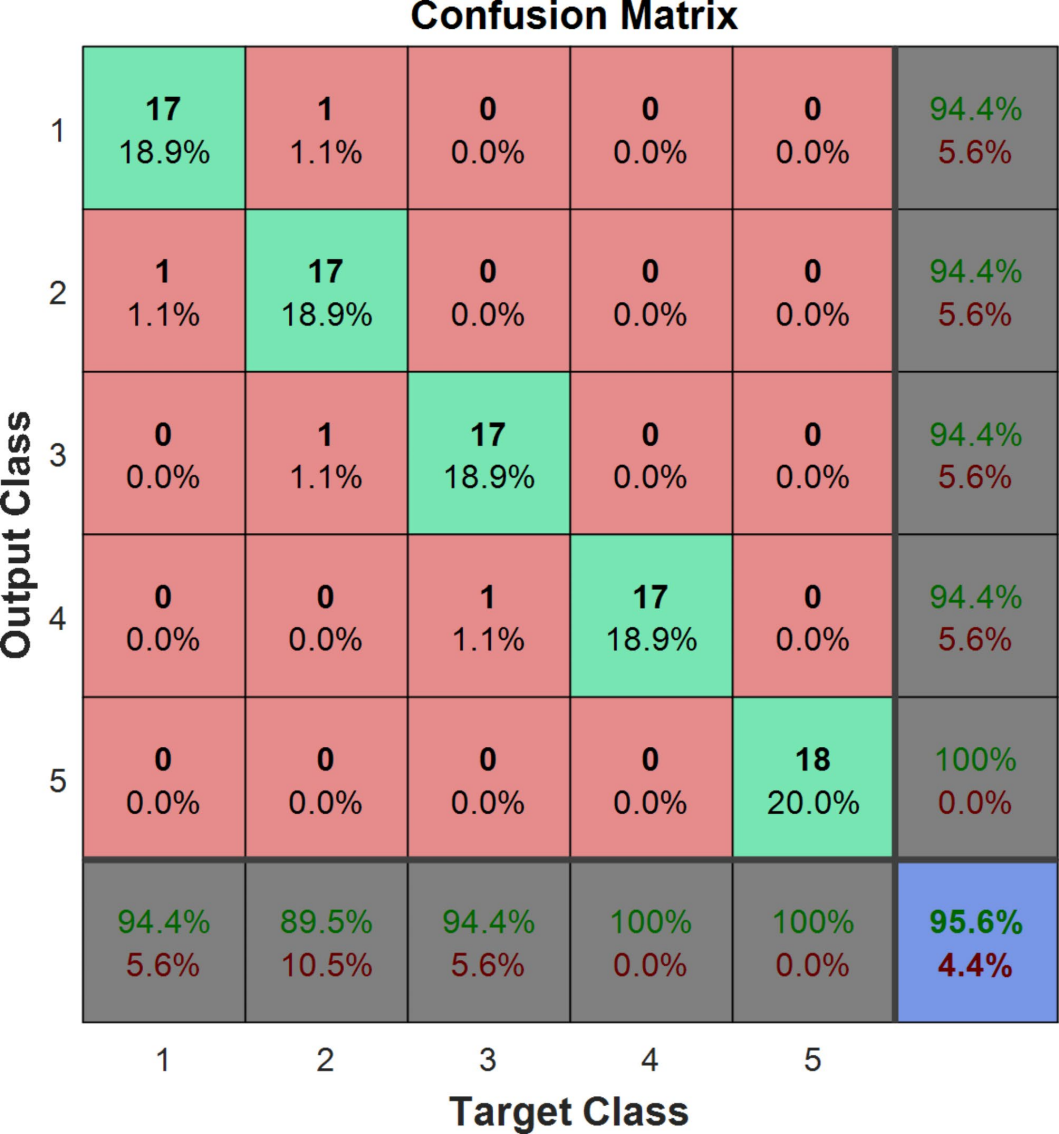
The confusion matrix of the optimal ANN classifier for detection of sea foam fraud in black pepper. Classes numbers from 1 to 5 indicate adulteration levels of 0, 5, 15, 30, and 50% levels, respectively.

The confusing matrix related to the optimal classifier based on the ANN method to detect the adulterated percentage of sea foam in black pepper has been given in Fig. 4. In this confusion matrix, class numbers 1, 2, 3, 4, and 5 means adulteration of sea foam in black pepper with 0, 5, 15, 30, and 50% levels. As shown in this figure, out of the total number of 90 samples with different percentages of sea foam fraud in black pepper, 86 samples have been correctly recognized. So, the accuracy of the optimal model was equal to 95.6%.

**Fig. 5:**
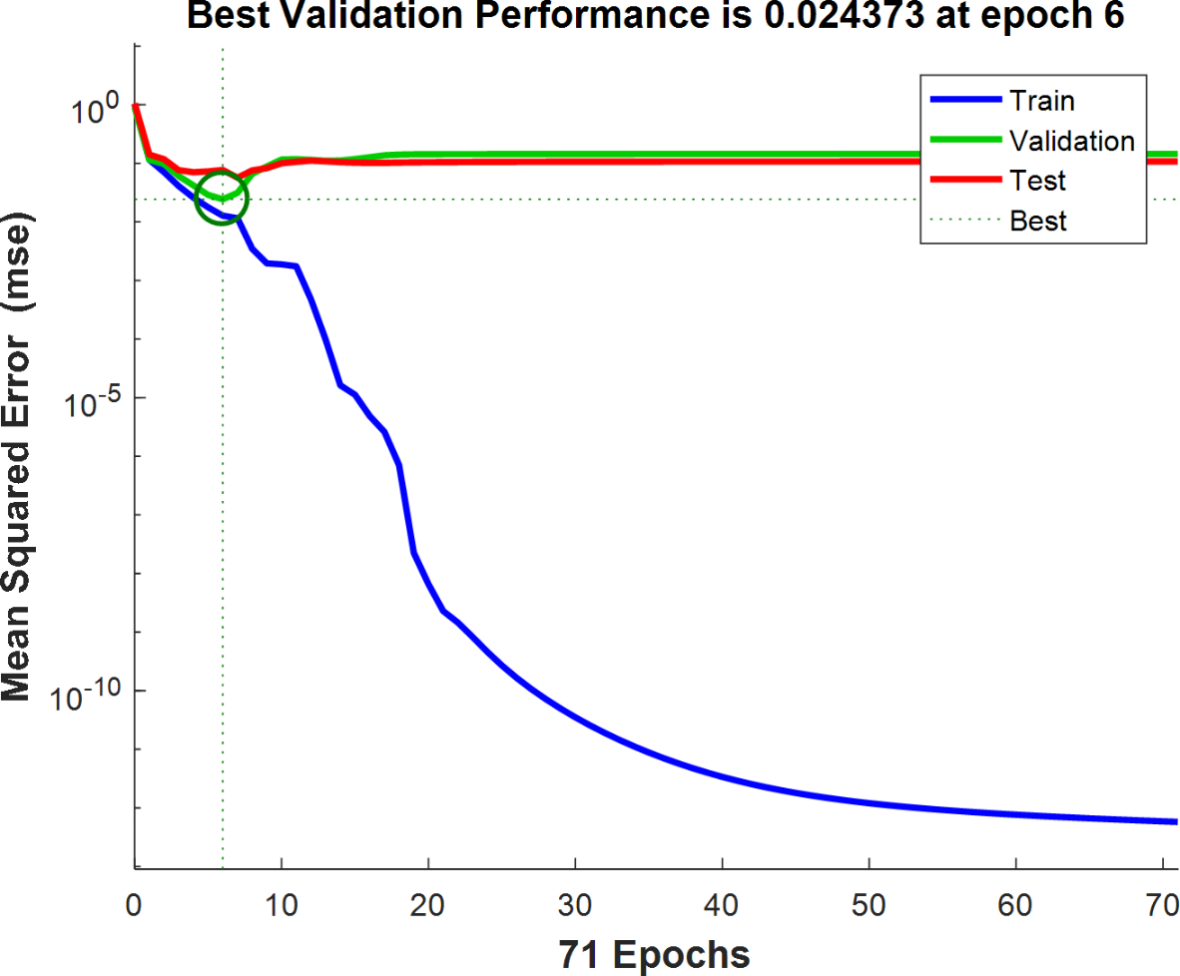
The validation performance of the optimum ANN classifier for detection of sea foam in black pepper.

The performance of the network in the validation phase for different epoch numbers has been shown in Fig. 5. As seen in this figure, the lowest validation error was equal to 24.37 × 10^− 3^ which was observed in epoch 6.

**Fig. 6:**
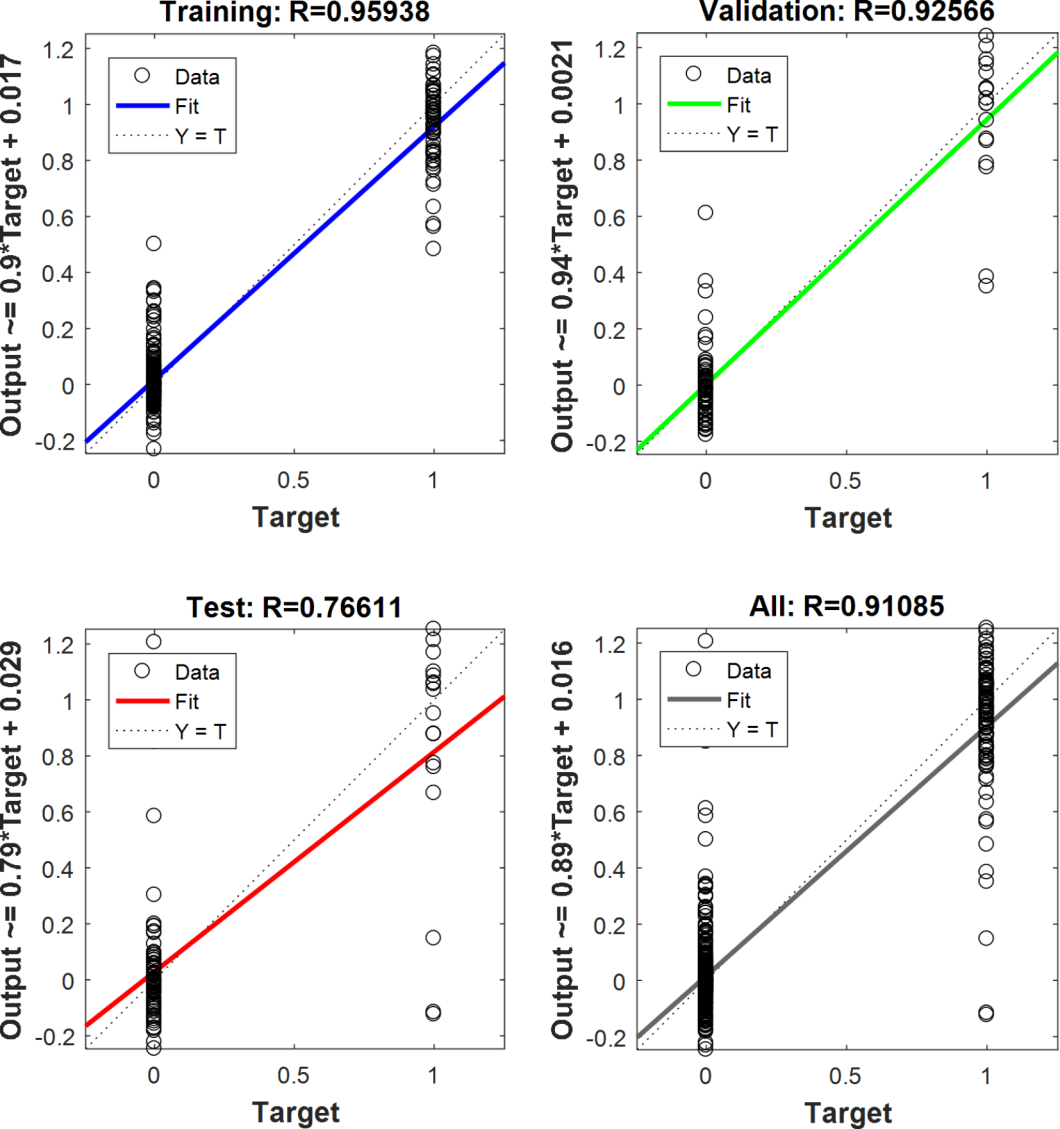
The regression results of the optimal ANN model in the classification of different fraud levels of black pepper.

The correlation coefficient of the optimal ANN model for training, validation, test, and total data has been shown in Fig. 6. The correlation coefficients of the optimal network for training, validation, test, and total data were equal to 95.94, 92.57, 76.61, and 91.09%, respectively. As can be seen in Fig. 6, only two targets exist in the diagrams because the targets in classification included 0 (incorrect output) and 1 (correct output) data.

**Fig. 7:**
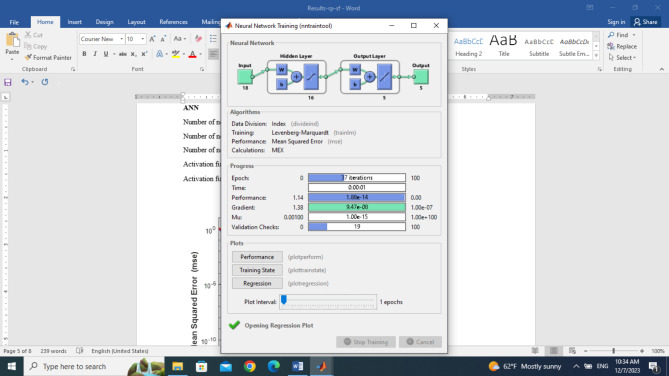
The optimal ANN structure for classification of different fraud levels of red pepper.

To find the best ANN model for classifying different samples of red pepper adulterated with sea foam, different structures were evaluated from 18-1-5 to 18-20-5, and the best model was selected based on validation performance, overall correlation, and overall correct classification rate. Figure 7 shows the classification of different samples of red pepper with sea foam fraud using the ANN method. The number of neurons in the input, hidden, and output layers was 18 (the number of efficient features), 16, and 5 (different fraud levels), respectively. The highest percentage of correct classification rate was obtained for the 18-16-5 structure.

**Fig. 8:**
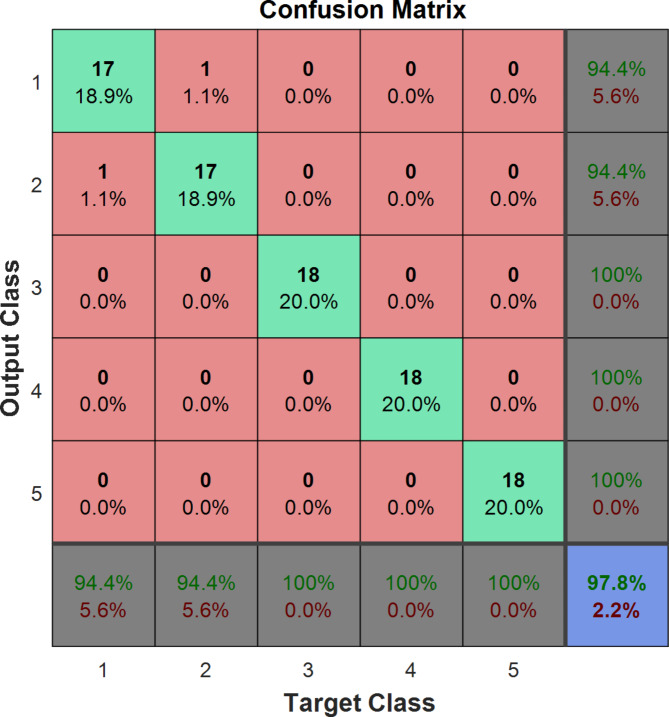
The confusion matrix of the optimal ANN model for the detection of sea foam fraud in red pepper. Classes numbers from 1 to 5 indicate are adulteration levels of 0, 5, 15, 30, and 50% levels, respectively.

**Fig. 9:**
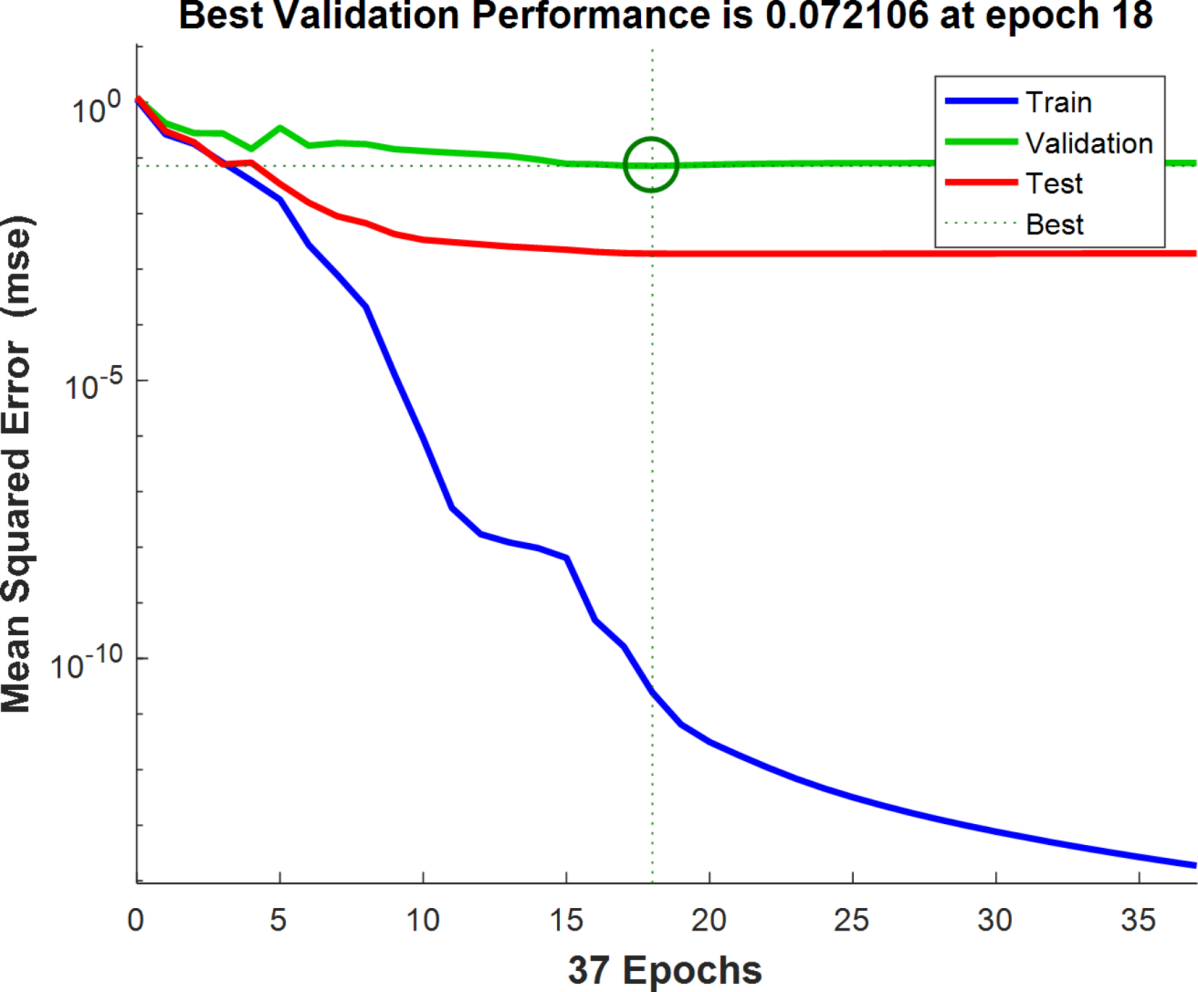
The performance of the optimal neural network classifier in the validation phase for the detection of wheat flour adulteration in red pepper.

Figure 8 shows the confusion matrix related to the optimal classification of red pepper with sea foam fraud using the artificial neural network method. Class 1, 2, 3, 4, and 5 mean the level of sea foam fraud in red pepper. Out of a total of 90 samples with different percentages of seabed adulteration in red pepper, 88 samples were correctly identified and only 2 samples were incorrectly classified. However, it can be said that the artificial neural network was able to classify the samples with a classification accuracy of 97.8%. As seen in Fig. 9, the lowest validation error was observed in epoch 18 as 72.11 × 10 − 3.

**Fig. 10:**
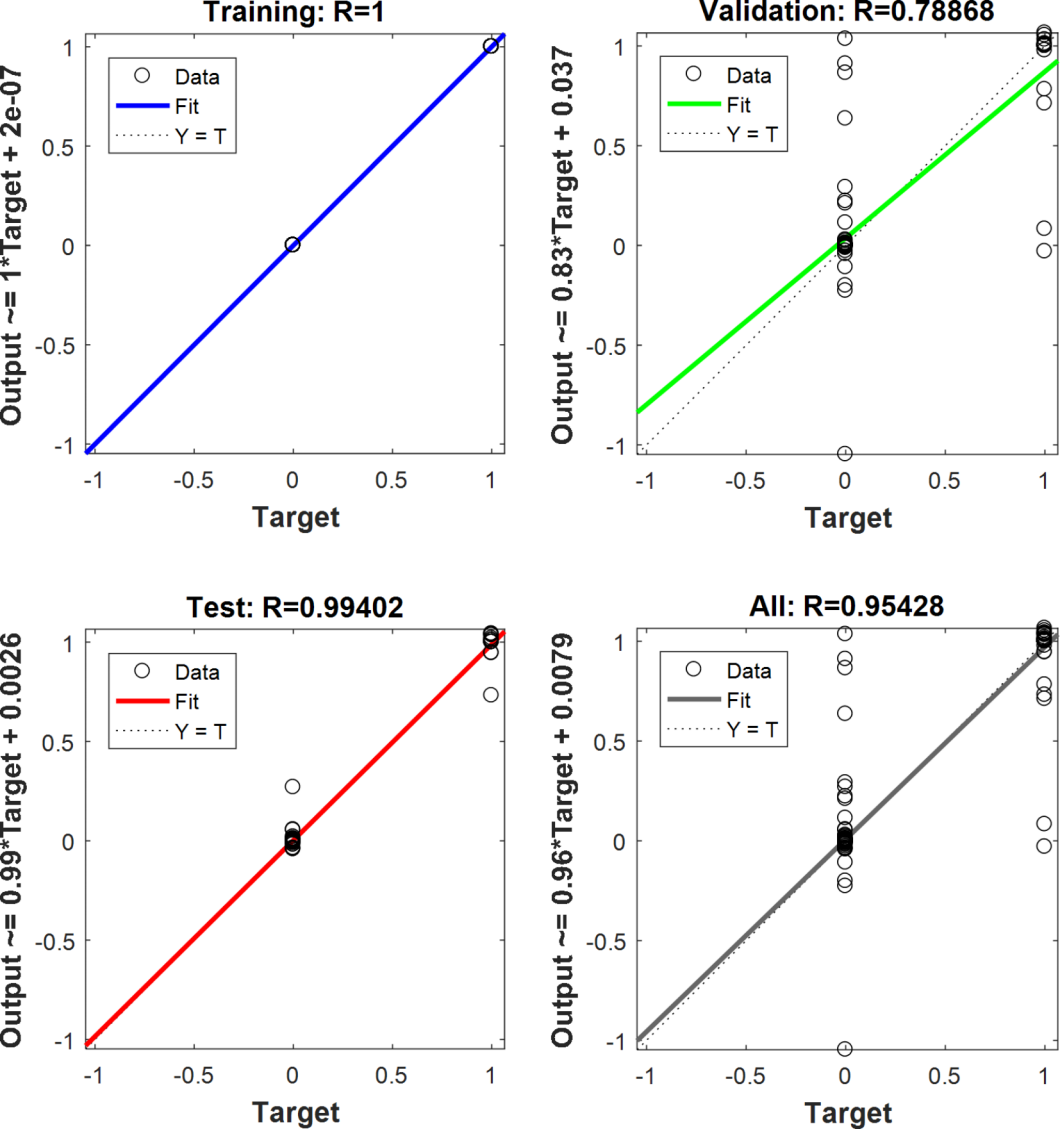
The correlation coefficients of the optimal ANN model for the detection of sea foam fraud in red pepper.

The correlation coefficients of the optimal ANN for training, validation, test, and total data were displayed in Fig. 10. As can be seen in Fig. 10, two targets exist in the diagrams because the targets in classification included only 0 (incorrect output) and 1 (correct output) data. It can be seen that the correlation coefficients of the optimal network in the stages were equal to 100.00, 78.87, 99.40, and 95.43%, respectively.

#### SVM classifier

**Fig. 11:**
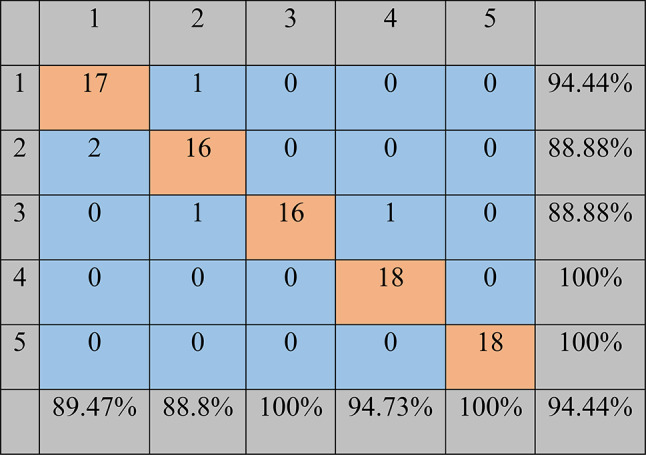
The confusion matrix of the SVM classifier with the one-against-one strategy for the classification of different fraud levels in black pepper. Class numbers of 1, 2, 3, 4, and 5 represent adulteration levels of 0, 5, 15, 30, and 50%, respectively.

Figure 11 shows the confusion matrix of the classifier model based on the support vector machine method with the one-to-one strategy to detect sea foam adulterant in black pepper. According to the results shown in the matrix, the classifier has correctly recognized all the samples in the fourth and third classes, which correspond to 30 and 50% fraud levels. In the first class, which was related to the pure black pepper samples, only one sample was wrongly misclassified and placed in class number 2 (5% fraud level). In the second and third classes, 5 and 15% fraud level, respectively, 16 out of 18 samples were correctly detected. In general, 85 out of 90 samples were correctly detected and 5 samples were misclassified. Therefore, the correct classification accuracy in this method was equal to 94.44%.

**Fig. 12:**
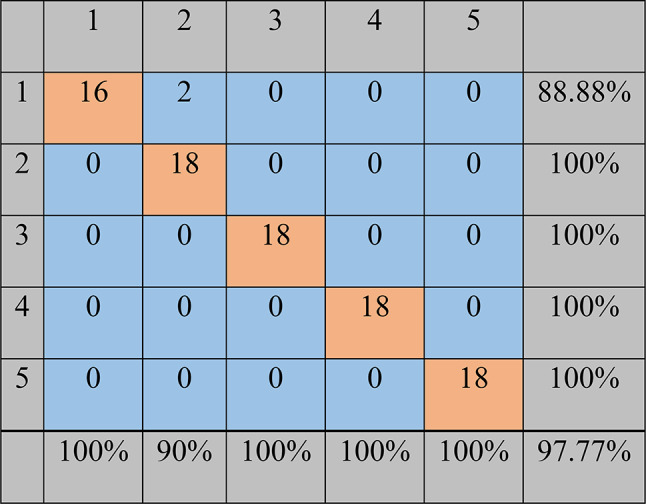
The confusion matrix obtained by the one-against-one support vector machine method for the classification of different fraud levels of sea foam in red pepper. Class numbers of 1, 2, 3, 4, and 5 represent adulteration levels of 0, 5, 15, 30, and 50%, respectively.

The confusion matrix of the classifier model based on the support vector machine method with the one-against-one strategy for detecting sea foam fraud in red pepper has been shown in Fig. 12. The classifier classified all samples of all fraud levels except for class number 1. It wrongly classified 2 samples out of 18 samples related to pure red pepper which placed in the 5% fraud level (class number 2). Finally, according to the confusion matrix, 88 out of 90 samples were correctly detected. Therefore, the accuracy of the method was equal to 97.77%.

#### Comparing the classifiers’ performance

The performance matrixes of the classifiers have been presented in Table 3. As seen in this table, the highest performance of black pepper has been obtained by the ANN. It had the highest amount of specificity, sensitivity, precision, and accuracy (98.89, 95.67, 95.56, and 98.22%, respectively). Reversely, the best performance for red pepper was obtained by the SVM method (99.46, 98.00, 97.78, and 99.11%, respectively).

**Table 3:** The mean values of the calculated performance metrics (%) of the classifiers.

Material	Method	Accuracy	Precision	Sensitivity	Specificity
Black pepper	ANN	98.22	95.56	95.67	98.89
	SVM	97.77	94.44	94.62	98.62
Red pepper	ANN	99.11	97.78	97.78	99.44
	SVM	99.11	97.78	98.00	99.46

The classification accuracies of the ANN method for detecting sea foam in black and red peppers in the present study were higher than those of the SVM method. The highest accuracies for the detection of sea foam levels in black and red peppers in the present research were 95.6 and 97.8%, respectively. These results were comparable with those presented in the previous research ^34,61,65^. The results of a research showed that the best network for detecting sunflower and rapeseed oils in olive oil had accuracies of 94.40 and 94.60%, respectively^11^. Different adulterations in milk were detected using the LED-based Vis-SWNIR photoacoustic spectroscopy system. The results of the research showed that the highest accuracy of the artificial neural networks classifier was 97.6%. The accuracy of detecting fraud in ginger powder (99.70%)^63^ was higher than those achieved in the present research. This is due to using deep learning but this technique requires a large number of samples and data. However, camera quality, camera calibration, and variations in lighting conditions and sample preparation may affect the results.

## Conclusions

In the present research, sea foam fraud in black and red pepper was detected. The fraud levels included 0, 5, 15, 30 and 50%. The visible images of different samples were acquired and processed to extract image features. Among the extracted features, efficient features were selected and classified using artificial neural network and support vector machine methods. The ANN had better results than the SVM method for the classification of different adulterant levels in black pepper with specificity, sensitivity, precision, and accuracy of 98.89, 95.67, 95.56, and 98.22%, respectively. Reversely, the best performance for red pepper was obtained by the SVM method with the metrics’ values of 99.46, 98.00, 97.78, and 99.11%, respectively.

Visible image processing combined with machine learning methods can be used to instead of laboratory-based fraud detection methods with higher speed and lower costs.

Although the present research aimed to evaluate the developed system based on visible image processing and machine learning techniques for detecting fraud in peppers, other products and adulterants can be studied in the future. To apply the developed system in the present research in industry, more samples can be used with different geographical locations, harvest seasons, and storage durations. Also, other adulterants in peppers can be studied to develop a strong system. Other data-analyzing methods and imaging techniques such as near-infrared hyperspectral imaging [24,48] can be used to achieve better results.

## Data Availability

The datasets used and/or analysed during the current study available from the corresponding author on reasonable request.
